# Association of Poor Social Support and Financial Insecurity with Psychological Distress of Chronic Kidney Disease Patients Attending National Nephrology Unit in Sri Lanka

**DOI:** 10.1155/2018/5678781

**Published:** 2018-05-16

**Authors:** Ramya Hettiarachchi, Chrishantha Abeysena

**Affiliations:** ^1^Community Medicine, Postgraduate Institute of Medicine, University of Colombo, Colombo, Sri Lanka; ^2^Department of Public Health, Faculty of Medicine, University of Kelaniya, Colombo, Sri Lanka

## Abstract

**Background:**

Chronic kidney disease (CKD) is associated with high morbidity and mortality. Hence, CKD patients are often in chronic psychological distress. The objective of the study was to describe factors associated with psychological distress of CKD patients attending National Nephrology Unit.

**Methods:**

A descriptive cross-sectional study was conducted among 382 CKD patients above 18 years of age applying systematic sampling. The data was collected using self-administered questionnaires to assess the psychological distress (GHQ-12), social support (SSQ6), coping strategies (BRIEFCOPE), pain (0 to 10 numeric pain rating scale), and physical role limitation due to ill health (SF36QOL). Sociodemographic and disease-related data were collected using an interviewer administered questionnaire and a data extraction sheet. Multiple logistic regression was applied for determining the associated factors. The results were expressed as adjusted odds ratio (AOR) and 95% confidence intervals (95% CI).

**Results:**

Percentage of psychological distress was 55.2% (95% CI: 48.4% to 62%). Poor social support (AOR = 1.81, 95% CI: 1.14–2.88), low satisfaction with the social support received (AOR = 4.14, 95% CI: 1.59–10.78), stages IV and V of CKD (AOR = 2.67, 95% CI: 1.65–4.20), presence of comorbidities (AOR = 2.38, 95% CI: 1.21–4.67), within one year of diagnosis (AOR = 2.23, 95% CI: 1.36–3.67), low monthly income (AOR = 2.26, CI: 1.26–4.06), higher out-of-pocket expenditure per month (AOR = 1.75, 95% CI: 1.75–1.99), and being a female (AOR = 2.95, 95% CI: 1.79–4.9) were significantly associated with psychological distress.

**Conclusions:**

More than half of the CKD patients were psychologically distressed. Factors such as financial and social support will be worth considering early because of their modifiability.

## 1. Background

Chronic kidney disease (CKD) is reported as a major global public health problem due to growing number of patients and heavy cost [1, 2]. Around 10% of global population suffers from CKD similar to the burden of diabetes [2, 3]. Significant number of new CKD patients is being reported throughout Sri Lanka since recent past. CKD is the 7th cause of death and 3500 deaths occurred due to kidney disease in Sri Lanka in year 2012 [4]. Therefore, more attention must be paid to improve the wellbeing and reduce the mortality of these patients [5].

The physical discomfort due to the illness as well as treatment modalities such as oral medications, hemodialysis, renal transplantation, dietary, and fluid restrictions hamper the psychological wellbeing of CKD patients. High prevalence of psychological issues associated with CKD led to the introduction of Psychonephrology [6, 7]. Researchers have identified female gender, living alone, loss of employment, high out-of-pocket expenditure and reduced economic productivity, multimorbidity, increasing severity of illness, and stages four and five of the disease as factors significantly associated with psychological distress among CKD patients [8, 9], while social support and coping strategies are known to reduce the distress [10, 11].

Improper adherence to medical advice leads to increased morbidity and mortality in CKD [12, 13]. Further, patients with end stage renal failure, a deadly incurable illness, need to undergo dialysis or a renal transplant for survival. This will further aggravate the psychological distress [14, 15].

Therefore, periodical assessment of psychological status of CKD patients is needed [7]. Psychological distress among CKD patients had not been assessed previously in Sri Lanka. Therefore, the aim of the study was to describe the factors associated with psychological distress of CKD patients attending National Institute of Nephrology Unit in Sri Lanka.

## 2. Methods

A hospital-based cross-sectional study was carried out at the National Institute of Nephrology Unit, Colombo, Sri Lanka, during April to September 2015. All the CKD patients registered in the clinic with evidence of chronic renal disease documented in a diagnosis card or in a clinic book participated in the study irrespective of cause and duration of illness. We excluded patients with depleted neurological state (Glasgow coma scale of <15), patients with a psychiatric illness.

A total final sample size of 420 was calculated with the critical value as 1.96 which corresponds to 95% confidence limit, precision as 5%, and an expected prevalence as 55% [16] with 10% nonresponse. Systematic sampling technique was used to enroll patients to the study. Source for the sampling frame was the clinic attendance register. Every third patient was invited to participate in the study until the total sample size was achieved.

The questionnaire comprised two sections. Section one was an interviewer administered questionnaire comprising variables on sociodemographic, economic, and disease-related factors. Section two was a self-administered questionnaire. It included a short form of social support questionnaire (SSQ6) to measure social support [17], BRIEFCOPE to assess coping strategies [18], 0–10 pain rating scale to assess pain [19], and GHQ 12 to assess psychological distress [20].

The SSQ6 consists of six items to measure perceived social support and satisfaction for the received support for each item. It was translated and culturally adapted. A panel of experts provided judgmental validity for the instrument. With regard to provision of perceived social support, the maximum number of people for each item was confined to nine while minimum was zero. SSQ6 score ranged from 54 to zero. Total SSQ6 score was categorized into two for bivariate analysis: as poor social support when SSQ6 score was ≤25th percentile (≤13 people to give support) and as high social support when SSQ6 score was >25th percentile (>13). Satisfaction for the received social support from people was measured by six-point Likert scale. Minimum satisfaction score was six and maximum satisfaction score was 36. Total satisfaction score was categorized into two at 75th centile for bivariate analysis. Score < 75th (≤22.5) was considered low satisfaction and ≥75th was considered as higher social satisfaction over received support.

BRIEF COPE [18] was validated to Sri Lanka [21], with a reliability of Cronbach's alpha of 0.75. It was scored with four-point Likert scale. Coping strategies were categorized into three in BRIEF COPE as dysfunctional coping, problem focused coping, and emotion focused coping. Total coping score was calculated for each category. Each coping score was subcategorized into three as low, moderate, and high at 15th and 85th centiles. Number of patients with moderate and high coping responses were amalgamated for bivariate analysis.

Financial insecurity was assessed in terms of per capita monthly income and out-of-pocket expenditure for health per month. We obtained per capita monthly income of CKD patients in Sri Lankan rupees as a continuous variable. Monthly income of less than 10,000 Sri Lankan rupees per month was defined as low monthly income. Out-of-pocket expenditure for health per month was calculated adding total expenditure for drugs, medical investigations, transport, bystanders, doctor charges, and treatments such as for dialysis. More than 6000 Sri Lankan rupees were defined as high out-of-pocket expenditure per month.

CKD patients suffer from joint pain and perceived bodily muscular skeletal pain. Therefore, we measured pain in a severity scale of zero to ten (severe pain) [19]. Score of ≥four was taken as the cut-off value which indicates significant pain interfering with activities [19]. The life events over the last three months were measured using an instrument containing eight items [22]. We assigned a minimum score of zero and maximum score of eight. If a patient had experienced at least one life event during the last three months, they were categorized under “stress group.”

Problems faced with the work and other regular daily activities due to the physical ill health were assessed with relevant questions (physical functioning component) extracted from the Short Form 36 quality of life questionnaire [23]. Minimum score was zero and maximum score was four. The total score of ≤1 was considered minimum physical role limitation and total score of four was considered as the maximum physical role limitation due to ill health.

Accurate knowledge on prognosis of CKD was assessed by inquiring whether they agree to the statement “CKD is a controllable disease and has to undergo renal transplant or dialysis at the end stage.” Perceived knowledge on prognosis of CKD was assessed by inquiring whether they agree to the statement “CKD is progressive and incurable or non-progressive and curable disease.”

General Health Questionnaire Item 12 (GHQ12) which was validated to Sri Lanka [20] was used to assess the psychological distress of CKD patients. The total GHQ score of ≥2 was considered as psychological distress.

A record sheet was used to collect secondary data such as serum creatinine, stage of CKD, treatment modality, and comorbidities from patient's clinic records. Pretest of the questionnaire was carried out among ten CKD patients. Eligible patients were invited to participate after their clinic activities. The questionnaire was given to patients in a separate room with a view to maintain privacy and confidentiality in the absence of any person other than the patient and the data collector.

Data was analyzed using SPSS version 16. Psychological distress of CKD patients was assessed with the univariate analysis expressed as percentages and confidence intervals. Bivariate analysis was applied for assessing the association between the factors and psychological distress of CKD patients. All variables at bivariate analysis with *P* value < 0.2 were eligible for multiple logistic regression analysis which was applied for controlling confounding factors. Results were expressed as adjusted odds ratios (OR) and 95% confidence intervals (CI).

Permission to conduct the study was obtained from the Director of the National Institute of Nephrology and from the respective consultant nephrologists. All eligible patients were explained regarding the study. Informed written consent was obtained from those who were eligible and willing.

## 3. Results

We invited 420 eligible CKD patients. Nonresponse rate was 9.1%. Therefore, 382 patients participated in the study. Mean age of the study population was 54.5 years (SD: +/−31.6). Majority of CKD patients were males (212, 55.5%), Sinhalese (329, 86.1%), and Buddhists (279, 73%) from Gampaha District (138, 36.1%), studied up to ordinary level examination (237, 62%), and in end stage renal failure (125, 32.7%).

### 3.1. Percentage of Psychological Distress

Response rate for the GHQ12 was 89.7% (377). Of them, 55.2% (CI: 54.7%–55.7%) had psychological distress. The percentage of psychological distress was higher among patients aged between 61 and 80 years (57.8%) and among females (62.7%), non-Sinhalese (66%), and non-Buddhists (64.6%) and who were in stage V of the illness (71.9%) (Table 1).

### 3.2. Factors Associated with Psychological Distress

As shown in Table 2, psychological distress of CKD patients was significantly associated with demographic and economic factors such as female gender, patients educated up to ordinary level, currently unemployed, out-of-pocket expenditure of >6000 rupees per month, and distance to the nephrology clinic from living place of >150 km.

Of all social and disease-related factors, duration of illness < 12 months, visiting the nephrology clinic > 1 per month, maximum physical role limitation due to ill health, perceived knowledge regarding prognosis of chronic kidney disease, poor social support, low satisfaction for the received social support, stages IV and V of the disease, currently undergoing dialysis, and having comorbidities (one or more) were significantly associated with psychological distress of CKD patients (Table 3).

A statistically significant association was observed between psychological distress of CKD patients with poor social support, less satisfaction over received social support, presence of comorbidities, out-of-pocket expenditure for health more than 6000 rupees per month, low monthly income, female gender, duration since diagnosis 12 months or less, and stages IV or V renal disease (Table 4).

## 4. Discussion

The present study found that 55.2% of CKD patients attending Maligawatta nephrology clinic suffered from psychological distress. The percentage of psychological distress among CKD patients was higher than the general population (5%–27%) as well as cancer patients (30%) [24, 25]. The present study finding was compatible with two other studies [9, 16]. Sumanathissa reported prevalence of psychological distress as 55% in the Anuradhapura nephrology clinic [16] in Sri Lanka. Another study conducted in Sweden to measure psychological distress among CKD patients revealed it as 53.5% [9].

The present study revealed that female CKD patients were almost three times more psychologically distressed than male CKD patients. According to Sumanathissa, depression was associated with female CKD patients in Anuradhapura nephrology clinic [16]. Similarly, Sfyrkou in Sweden reported that women have high psychological distress compared to men [9].

In the present study, CKD patients who had a low monthly income were more likely to be distressed than CKD patients who earned adequate monthly income. This finding was consistent with two other studies, a 12-year cohort study done by Orpana et al. in USA [26] and another in UK [27] by Kosidou et al., which have detected low income as significantly associated with psychological distress when adjusted for sociodemographic characteristics and baseline health characteristics.

CKD patients who spend more than >6000 rupees as out-of-pocket expenditure for health per month were significantly distressed than CKD patients who spend ≤6000 rupees per month. Some CKD patients of the study sample received only limited number of free dialysis sessions. Thereafter, they had to bear the cost for the rest of dialysis sessions. And some of the patients from faraway places get their investigations done from private sector as well. However, findings of a study done in Australia [28] in 2015 were contrary to ours. High out-of-pocket expenditure was not associated with depression among CKD patients when adjusted for confounding factors such as household income, occupation, and government concessions.

In our study, presence of one or more comorbidities was significantly associated with psychological distress of CKD patients. A hospital-based study done by Jayasinghe on CKD patients in Badulla found that poor mental functioning score was significantly associated with the presence of one or more comorbidities [22]. A similar finding has been reported by Sfyrkou in Sweden [9].

It was noted that CKD patients in stages IV and V were having significantly higher psychological distress compared to CKD patients in stage one to three. Similar findings have been described by Chiang et al. in 2013 [8]. A meta-analysis, carried out by Palmar et al., found that stage five CKD patients were having significant depressive symptoms compared to patients in stage one to four [29].

Emotions sharing, financial aid, and any kind of support for the diseased patient relieve psychological distress. Therefore, social support has a public health importance because this is a modifiable factor. In the present study, social support was measured in two ways: number of people available to give support and the satisfaction over received support. Poor social support in both the domains was significantly associated with high psychological distress. In the study by Patel et al. among end stage renal disease patients, a higher depression score with perceived lower social support was found [10]. A study done by Kimmel et al. describes that social support leads to good mental health score and even increases the survival as well [7, 15]. Cukor et al. describe that social support improves the quality of life by acting as a protective factor against psychological ill health and stress [6].

## 5. Conclusion

More than half of the CKD patients (55.2%) were psychologically distressed. More attention should be paid to the factors associated with psychological distress. Activities should be taken especially on the modifiable factors like improvement of quality and quantity of social support, reduction of out-of-pocket expenditure, establishment of social security system, and increase of the income level of CKD patients. Because the study is a hospital-based one extrapolation of results to the broader population should be done with caution.

## Figures and Tables

**Table 1:** Percentage of psychological distress according to selected factors.

Factors	Sample size	Psychological distress		95% confidence interval
		Number	Percentage	
Age in years				
18–40	60	33	55.0	52.1 to 57.9
41–60	149	87	58.4	57.3 to 59.3
61–80	162	85	52.5	51.4 to 53.6
≥81	6	3	50.0	17.3 to 52.7
Sex				
Male	166	104	49.3	48.4 to 50.2
Female	211	104	62.7	61.8 to 63.6
Ethnicity				
Sinhalese	327	175	53.5	54.1 to 52.9
Non-Sinhalese	50	33	66.0	62.8 to 69.2
Religion				
Buddhists	278	144	51.8	52.5 to 51.1
Non-Buddhists	99	64	64.6	66.1 to 63.1
Marital status				
Unmarried	48	25	52.1	48.2 to 56.0
Ever married	329	183	55.6	55.0 to 56.1
Stage of the illness				
Stage 01	42	21	50.0	54.7 to 45.3
Stage 02	58	22	37.9	33.6 to 42.2
Stage 03	114	58	50.2	52.6 to49.2
Stage 04	71	42	59.2	56.9 to 61.5
Stage 05	89	64	71.9	70.5 to 73.3

Total	377	208	55.2	54.8 to 55.8

**Table 2:** Unadjusted odds ratios of demographic and economic factors and psychological distress.

Factors	Psychological distress		Odds ratio	95% confidence interval	*P* value
	Yes *n* (%)	No *n* (%)			
Age: <60 years	121 (58.2)	89 (52.7)	1.25	0.83 to 1.88	0.28
Sex: females	104 (50.0)	61 (36.7)	1.73	1.14 to 0.64	0.01
Ethnicity: Sinhala	175 (84.1)	152 (89.9)	0.59	0.31 to 1.10	0.09
Province of living: western	140 (67.3)	120 (71.0)	0.84	0.54 to1.30	0.44
Educational status: ≤O/L	89 (42.8)	53 (31.4)	1.64	1.07 to 2.50	0.02
Religion: Buddhists	144 (69.2)	134 (79.3)	0.58	0.37 to 0.94	0.03
Marital status: unmarried	25 (12.0)	23 (13.6)	0.87	0.47 to 1.6	0.64
Living alone	24 (11.5)	22 (13.0)	0.87	0.47 to 1.61	0.64
Currently employed	121 (58.7)	73 (43.2)	1.87	1.24 to 2.81	0.003
Low monthly income	52 (25.0)	28 (16.6)	1.68	1.0 to 2.8	0.05
High out-of-pocket expenditure	108 (51.9)	67 (39.6)	1.64	1.09 to 2.48	0.01
Distance to nephrology unit from residence: >150 Km	32 (15.4)	13 (7.7)	2.18	1.1 to 4.3	0.02

**Table 3:** Unadjusted odds ratios of social and disease related factors and psychological distress.

Factors	Psychological distress		Odds ratio	95% confidence interval	*P* value
	Yes *n* (%)	No *n* (%)			
Duration of illness since diagnosis: <12 months	83 (39.9)	48 (28.4)	1.67	1.08 to 2.58	0.02

Frequency of hospital visits per month: >once a month	75 (36.1)	41 (24.3)	1.76	1.12 to 2.76	0.01

Presence of maximum physical role limitation due to ill health	146 (70.2)	99 (58.6)	1.66	1.08 to 2.55	0.02

Significant pain interfering with activities	104 (50.0)	68 (40.2)	1.48	0.98 to 2.24	0.06

Perceived knowledge regarding the prognosis of CKD	106 (51.0)	56 (33.1)	2.1	1.38 to 3.19	0.001

Accurate knowledge on prognosis of CKD	148 (71.2)	115 (68.0)	1.15	0.74 to 1.8	0.51

Social support					
Number of people giving support ≤13 (poor social support)	124 (59.6)	72.0 (42.6)	1.99	1.31 to 3.0	0.001
Satisfaction over social support received ≤22.5 (low satisfaction)	31 (14.9)	7 (4.1)	3.94	1.68 to 9.21	0.001

Disease severity: stages IV and V	129 (62.9)	66 (39.1)	2.55	1.68 to 3.87	0.001

Treatment modality: dialysis	68 (32.7)	20 (11.8)	3.61	2.09 to 6.27	0.001

Presence of comorbidities	183 (88.0)	135 (79.9)	1.84	1.05 to 0.23	0.03

Experience of at least one life event	136 (65.4)	99 (68.6)	1.34	0.88 to 2.03	0.17

Coping strategies					
Dysfunctional coping: low	13.0 (6.5)	11.0 (6.9)	0.96	0.42 to 2.21	0.93
Problem focused coping: low	9.0 (4.4)	6.0 (3.6)	1.24	0.43 to 3.54	0.93
Emotion focused coping: low	174 (83.7)	133 (78.7)	1.38	0.82 to 2.33	0.23

**Table 4:** Adjusted odds ratios of the factors associated with psychological distress of CKD patients.

Factors	B	Standard error	OR	95% CI for OR		*P* value
Poor social support	0.59	0.24	1.81	1.14	2.88	0.01
Low satisfaction over received social support	1.42	0.49	4.13	1.59	10.78	0.004
Presence of at least one comorbidity	0.87	0.34	2.38	1.21	4.67	0.01
High out-of-pocket expenditure	0.56	0.24	1.75	1.09	2.82	0.001
Low monthly income	0.81	0.3	2.26	1.26	4.06	0.007
Sex: female	1.09	0.26	2.96	1.79	4.9	0.006
Duration of illness since diagnosis ≤12 months	0.80	0.25	2.23	1.36	3.67	0.001
Distance to nephrology unit from residence: >150 Km	0.71	0.38	2.03	0.97	4.25	0.06
Presence of maximum physical role limitation due to ill health	0.41	0.25	1.50	0.93	2.43	0.1
Disease severity: stages IV and V	0.98	0.24	2.66	1.65	4.29	0.001

B = beta coefficient; OR = odds ratio; and CI = confidence interval.
